# Clinical Significance of SOX10 Expression in Human Pathology

**DOI:** 10.3390/cimb45120633

**Published:** 2023-12-15

**Authors:** Hisham F. Bahmad, Aran Thiravialingam, Karthik Sriganeshan, Jeffrey Gonzalez, Veronica Alvarez, Stephanie Ocejo, Alvaro R. Abreu, Rima Avellan, Alejandro H. Arzola, Sana Hachem, Robert Poppiti

**Affiliations:** 1 The Arkadi M. Rywlin M.D. Department of Pathology and Laboratory Medicine, Mount Sinai Medical Center, Miami Beach, FL 33140, USA; robert.poppiti@msmc.com; 2 Herbert Wertheim College of Medicine, Florida International University, Miami, FL 33199, USA; athir006@med.fiu.edu (A.T.); ksrig002@med.fiu.edu (K.S.); jgonz1074@med.fiu.edu (J.G.); socej001@med.fiu.edu (S.O.); aabre070@med.fiu.edu (A.R.A.); ravel001@med.fiu.edu (R.A.); aarzo006@med.fiu.edu (A.H.A.); 3 Department of Anatomy, Cell Biology, and Physiological Sciences, Faculty of Medicine, American University of Beirut, Beirut 1107, Lebanon; sih12@mail.aub.edu; 4 Department of Pathology, Herbert Wertheim College of Medicine, Florida International University, Miami, FL 33199, USA

**Keywords:** SOX10, neural crest cells, melanoma, neuroectodermal tumors, mesenchymal tumors

## Abstract

The embryonic development of neural crest cells and subsequent tissue differentiation are intricately regulated by specific transcription factors. Among these, *SOX10*, a member of the *SOX* gene family, stands out. Located on chromosome 22q13, the *SOX10* gene encodes a transcription factor crucial for the differentiation, migration, and maintenance of tissues derived from neural crest cells. It plays a pivotal role in developing various tissues, including the central and peripheral nervous systems, melanocytes, chondrocytes, and odontoblasts. Mutations in *SOX10* have been associated with congenital disorders such as Waardenburg–Shah Syndrome, PCWH syndrome, and Kallman syndrome, underscoring its clinical significance. Furthermore, SOX10 is implicated in neural and neuroectodermal tumors, such as melanoma, malignant peripheral nerve sheath tumors (MPNSTs), and schwannomas, influencing processes like proliferation, migration, and differentiation. In mesenchymal tumors, SOX10 expression serves as a valuable marker for distinguishing between different tumor types. Additionally, SOX10 has been identified in various epithelial neoplasms, including breast, ovarian, salivary gland, nasopharyngeal, and bladder cancers, presenting itself as a potential diagnostic and prognostic marker. However, despite these associations, further research is imperative to elucidate its precise role in these malignancies.

## 1. Introduction

During embryonic stages, the formation of the primitive neural crest gives rise to diverse neural structures. These neural crest cells undergo differentiation into various tissues, a process regulated by specific transcription factors with varying expression levels. The SRY-related HMG box, also known as the *SOX* gene, plays a multifaceted role in differentiating embryological and biological processes among neural crest cells. The *SOX* gene family comprises eight subfamilies. 

**Figure 1:**
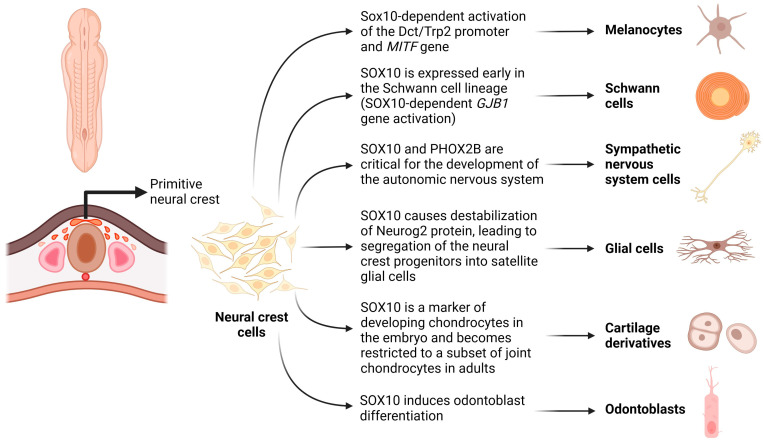
Schematic demonstrating the role of SOX10 in embryonic development, facilitating neural crest cell differentiation, and giving rise to several sublineages, including melanocytes, Schwann cells, sympathetic nervous system cells, glial cells, odontoblasts, and cartilage derivatives (chondrocytes). Created with BioRender.com (2023).

Within the SoxE subfamily, SOX10 emerges as a distinctive transcription factor that significantly contributes to the enhancement of differentiation, migration, and maintenance of tissues derived from neural crest cells. Initially expressed in the dorsal neural tube, SOX10 guides the differentiation of tissues within the peripheral nervous system [1,2]. This gene’s pivotal role in embryonic development facilitates neural crest cell differentiation, giving rise to several sublineages, including the arachnoid and pia mater, melanocytes, odontoblasts, tracheal cartilage, laryngeal cartilage, and Schwann cells (Figure 1).

The *SOX10* gene is located on chromosome 22q13, encoding the SOX10 protein with an open reading frame consisting of 466 amino acids and a weight of 51 kDa [3]. The protein possesses a highly conserved dimerization domain at its N-terminus within the SoxE subfamily. Comprising 40 amino acids, this region facilitates the protein’s dimerization ability for binding target genes. Adjacent to the N-terminus is the high mobility group (HMG) domain, spanning 79 amino acids and maintaining a consistent structure across all SOX family members. This domain, characterized by three alpha helices forming an L-shape, is designed for binding DNA sequences within the minor groove, specifically containing the nucleotide sequence of C[A/T]TTG[A/T][A/T]. This binding modulates DNA molecules, creating a compatible structure for active transcriptional complexes [3].

Within the domain, an intron and K2 domain are present, along with a nuclear localization and export signal [3]. The K2 domain functions as a promoter-specific transactivation domain, TAM (transactivation domain in the middle of the protein), crucial for SOX10 expression in the peripheral nervous system [4]. On the opposite end of the protein, in the C-terminal region, 66 amino acids are located, marked by a high expression of serine, prolines, and glutamine sequences [5]. This C-terminus is essential for SOX10′s interaction with specific binding targets during tissue differentiation, facilitated by a transactivation domain (TA or TAC) [3].

The distinctive composition of SOX10 enables it to exist as a monomer or dimer, exerting influence on various DNA binding targets with differing affinities. Beyond this dual functionality, SOX10 also serves as a nucleocytoplasmic shuttle protein for transcriptional activation, potentially binding to cis elements on target genes to regulate their expression [6,7]. These specific functions are intricately regulated through the modification and expression of SOX10, involving various signal transduction pathways such as Wnt, BMPs, and FGFs pathways [2,3,8].

Wnt signaling, in particular, plays a crucial role in neural crest formation. Decreased levels of Wnt signaling inhibit neural crest formation, underscoring its necessity in this developmental process. A study demonstrated that blocking Wnt using a second messenger resulted in the suppression of SOX10 expression [2]. Moreover, over a dozen transcription factors bind to the N-terminus of the SOX10 HMG domain, regulating its transcriptional activity [9]. *SOX9* and *Slug* are implicated in the regulation of SOX10, showing their necessity in neural crest cell development. Manipulating Slug and Sox9 expression, whether wild type or mutant, resulted in high or absent SOX10 expression, suggesting a mutual relationship between Slug and SOX10 [2].

Various modifications, including phosphorylation, acetylation, SUMOylation, and methylation, have been identified in different amino acid residues of SOX10. SUMOylation at three lysine residues (K55, K246, and K357) represses the transcriptional activation of target genes crucial for cell development and maintenance, such as *MITF* in melanocytes and *GJB1* in Schwann cells [10]. Additionally, phosphorylation of Ser24 and Thr240, two highly conserved sites within the SoxE family, has been associated with melanoma [11].

SUMOylation of SoxE proteins is integral to the development of the inner ear. A yeast two-hybrid screen identified UCB9 and SUMO-1 in SoxE proteins, including SOX10 and SOX9, crucial for inner ear regulation. Both SOX10 and SOX9 feature two conserved SUMOylation sites—one at the N-terminal of an E1 domain and the other at the C-terminal of the activation domain. Specifically, SOX10 undergoes SUMOylation at K44 and K333, at the N-terminus and activation domain, respectively, in addition to other conserved sites [12]. SUMOylation may also occur at K55 and K357 sites within the SOX10 due to their involvement in the interaction of UBC9 and SOX10 [13]. Consequently, the absence of a SUMOylated site may indicate the non-expression of a lysine residue in a SOX10 variant.

The expression of SOX10 varies in response to SUMOylation or the absence of necessary residues in SOX9 [12]. This evolved ability of SOX10 to undergo SUMOylation plays a pivotal role in regulating the protein, enabling it to modulate distant proteins, up- and downregulate various cellular functions, and modify protein complex interactions.

Given the highly conserved expression of SOX10 within neural crest cells and their derivatives, the presence of mutated variants can result in a spectrum of severe to lethal diseases. Over half of the variations within the SOX10 family result from truncations. The remaining variants include non-truncating, missense, in-frame insertions or deletions, and partial copy number variants. Missense mutations typically cluster in the HMG domain [3]. These mutations can lead to conditions such as deafness, dysregulation of the peripheral and central nervous systems, embryonic lethality, colonic issues, and various neoplasms.

In cases of sensorineural hearing loss, various *SOX10* mutations may lead to the agenesis or hypoplasia of semicircular canals and enlarged vestibules. Imaging modalities, including computed tomography (CT) and magnetic resonance imaging (MRI), have revealed a connection between *SOX10* mutations and the absence or hypoplasia of these structures [3]. These malformations associated with *SOX10* mutations have also been linked to dysregulation of *WNT1* (regulating cell fate), *KCNQ4* (potassium voltage-gated channel), *STRC* (stereocilin, associated with the hair bundle of the ear), and *PAX6* (paired box 6) [3].

Considering the crucial role of *SOX10* in myelin-containing glial cells, various mutations have been identified. Two frameshift mutations within the carboxy-terminal, resulting in truncations (*SOX10Dom* and *SOX10*-*59*), have been associated with dominant megacolon and Waardenburg–Hirschsprung disease [14]. A group of disorders collectively labeled as PCWH (peripheral demyelinating neuropathy, central demyelinating leukodystrophy, Waardenburg syndrome, and Hirschsprung disease) result from variants within the nervous system. Clinical presentations may include delayed motor and cognitive development, cerebral palsy, ataxia, spasticity, congenital nystagmus, hyporeflexia, distal sensory impairments, and distal muscle wasting [3]. Signs of Kallmann syndrome (KS) have also been observed in Waardenburg syndrome, suggesting that KS may result from *SOX10* mutations. KS manifests with hypogonadotropic hypogonadism and anosmia. Many patients with KS may also present with hearing deficits and harbor *SOX10* mutations [4]. The physiological basis of this disorder in relation to *SOX10* is believed to involve the dysregulation of GnRH (gonadotropin-releasing hormone) as it travels through the neurons of the peripheral olfactory nerve, up to and through the olfactory bulb [3].

*SOX10* plays a crucial role in the embryogenesis of neural crest cells, and deviations from its normal function can give rise to various congenital disorders. However, the impact of *SOX10* variants extends beyond developmental disorders, contributing to the initiation and progression of different cancers due to its involvement in numerous tissues.

SOX10 expression has been identified in various cancer types, including breast tumors, glioma, glioblastoma, salivary adenoid cystic tumors, melanoma, and hepatocellular carcinoma. Intriguingly, *SOX10* exhibits dual roles in these tumors, acting as a tumor suppressor and promoter. For instance, it functions as an oncogene in hepatocellular carcinoma and nasopharyngeal carcinoma while exerting tumor-suppressive effects in gastrointestinal neoplasms. Urothelial carcinoma shows an overexpression of *SOX10*, indicating its role as a tumor promoter [15]. The significance of SOX10 expression becomes evident when comparing its levels in different bladder cancers to normal bladder tissue [16].

**Figure 2:**
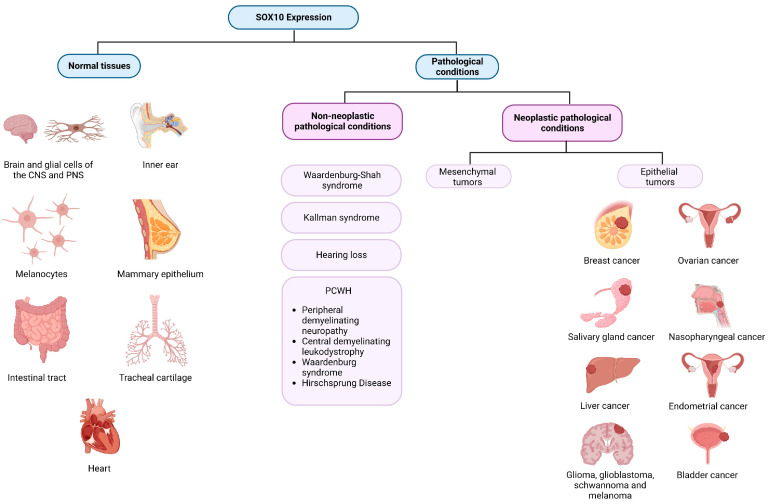
SOX10 expression in normal tissues and across different pathological conditions. Created with BioRender.com (2023).

These varied expressions underscore the need to study SOX10’s role and levels in both normal and pathological tissues. This comprehensive understanding is crucial for unraveling its precise role in cell biology and appreciating its clinical significance (Figure 2).

## 2. SOX10 Expression in Normal Tissues

The differentiation of various tissues from neural progenitor crest cells involves distinct processes. SOX10 expression remains elevated in tissues such as the brain, inner ear, intestinal tract, tracheal cartilage, and heart. In the early embryonic development of the inner ear, SOX10 shows high expression, gradually declining as hair cells mature. At this stage, SOX10 becomes specific to supporting cells, and an inability to express either SOX10 or SOX9 may result in the development of an enlarged or cystic otocyst [12,17,18].

Conversely, lower levels of SOX10 expression are observed in the prostate, testis, bladder, pancreas, and stomach [19]. During peripheral nervous system development, some neurons lose SOX10 expression while all mature glial cells maintain its expression. In the central nervous system, oligodendrocytes exhibit a high level of SOX10 expression. Similarly, melanocytes heavily rely on SOX10 for their specialization, maturation, and maintenance [2]. 

### 2.1. SOX10 Expression within the Peripheral Nervous System

Within the peripheral nervous system, *SOX10* plays a pivotal role in facilitating the differentiation of both Schwann cells and glial cells, employing distinct biochemical processes in each cell type. In Schwann cell development, SOX10 directly targets the protein zero (*P0*) gene coding region, a myelin gene exclusively expressed in Schwann cells, tightly regulated by *SOX10* [14].

Analysis of mouse embryos with mutated binding sequences on *P0* for *SOX10*, compared to those with normal binding sequences, revealed robust SOX10 expression in mature Schwann cells with high P0 expression. This expression was further intensified when a *SOX10* induction signal was introduced into these sequences, resulting in a ten-fold increase in P0 expression [14]. 

Neurogulin-1 has been identified as a key player in controlling the differentiation of neural crest cells into glia via the activation of ErbB receptors [20]. The absence of interaction between Neurogulins binding to the EGF receptor tyrosine kinase, ErbB3, has been associated with developmental defects in neural crest cells and their derivatives. The relationship between *SOX10* and ErbB3 was investigated using the tet-on system, inducing SOX10 expression, leading to a significant increase in ErbB3 expression. However, whether this effect was direct or indirect remained unclear. Supporting this relationship, *SOX10* mutant variations were found in ErbB3 mutant mice [20]. 

It is important to note that in certain cells, there was a high expression of SOX10 coupled with a low expression of P0, particularly in non-myelinating cells. This suggests that *SOX10* typically does not function independently but, instead, interacts with different protein complexes. In unmyelinated Schwann cells, the downregulation of myelination may be attributed to *SOX10*’*s* regulation of various transcription factors, including *SOX5*, *SOX6*, *NOTCH1*, *HMGA2*, *HES1*, *MYCN*, *ID4*, and *ID2*. These regulators were found to oppose the process of myelination within Schwann cells [21]. Furthermore, in an experimental study on SOX10 expression within mammary glands, mouse embryos were manipulated to be homozygous dominant knockout for *SOX10*. In these specific mice, death was quickly encountered, in addition to the complete absence of Schwann cell production [22].

### 2.2. SOX10 Expression within the Inner Ear

Moving beyond the peripheral nervous system to the inner ear, there is meticulous regulation of *SOX10* and *SOX9*, which is crucial for normal development. During gastrulation and neural crest development, SOX10 is expressed in the otic vesicle, reaching its peak around stage 25 [2]. Both SoxE proteins, SOX10 and SOX9, undergo SUMOylation at different lysine residues and two conserved sequences [12]. The regulation of these modifications may have subsequent consequences, leading to progressive hearing loss. 

SOX10 exhibits high expression in the otic vesicle from E9.5 onward until it becomes exclusively expressed in the supporting cells later in development. This sustained expression of SOX10 facilitates the maintenance of cochlear progenitors during the development of the organ of Corti and the otocyst [3]. 

### 2.3. SOX10 Expression in Melanocytes

The precise expression of SOX10 in melanocytes is indispensable for gene regulation within these cells. Before melanocyte development, SOX10 is highly expressed in the neural crest region, initially across all axial areas and later progressing to the expression only in the truncal region. Overexpression of SOX10 at this stage results in high expression in the Slug domain, both playing a role in the development of pigmented melanocytes [3].

Through a complex network, *SOX10* collaborates with *PAX3* to activate *MITF*, enhancing its expression. Increased *MITF*, in turn, works with *SOX10* to promote *DCT*/*TRP2* expression. Dominant *SOX10* mutant mice exhibit a decrease in melanocyte markers Dct/Trp2, underscoring the pivotal role of *SOX10* in pigment production [10]. Consistent with these cell markers, it has been shown that mutant *SOX10* or low expressions lead to a proportional decrease in markers Trp2, c-kit, and Mitf [3]. The varying levels of these markers depend on the stage of melanocyte development within the embryo, starting with nonpigmented melanoblasts and eventually transitioning to melanocytes. It has been demonstrated that SOX10 could produce pigment at injected sites, while Slug alone could not [3]. 

### 2.4. SOX10 Expression in the Mammary Epithelium

An exception to the typical expression of SOX10 in neural crest cell derivatives is observed in the mammary epithelium, which originates from the ectoderm. The mammary gland houses epithelium that bifurcates into the ductal epithelial tree during puberty. The mammary epithelium undergoes dynamic changes in growth due to hormonal stimulation during puberty, pregnancy, lactation, and menopause. SOX10 expression in these cells initiates prenatally during the development of stem cells.

Within these stem cells, *SOX10* responds to *FGF* signaling, facilitating their progression to mesenchymal tissue. A study manipulating mice embryos analyzed the effects of homozygous, heterozygous *SOX10* knockouts, and wild type. Both heterozygous and homozygous knockout mice exhibited decreased mammary branching growth development. Furthermore, postnatal mammary development revealed that these adult mice were unable to lactate after pregnancy.

Continuing through the female reproductive process, mice were further analyzed during involution. Compared to wild-type mice, knockout mice started with substantially fewer epithelial cells in the mammary glands. However, during involution, the epithelial cell count decreased significantly more in wild-type mice. These findings suggest the involvement of *SOX10* throughout the entire process, including the involution of expanded mammary epithelia. Although *SOX10* may play a crucial role in this process, the presence of mammary growth indicates that *SOX9* and *SOX10* may work synergistically, with *SOX9* contributing to the absence of *SOX10* [22].

## 3. SOX10 Expression in Non-Neoplastic Pathological Conditions

### 3.1. SOX10 in Waardenburg–Shah Syndrome

*SOX10* mutations have been implicated in disrupting neural crest development, leading to a diverse range of clinical phenotypes. The association of the *SOX10* gene with congenital disorders was initially recognized in the context of Waardenburg–Shah syndrome, a subtype of Waardenburg syndrome (WS), also known as Waardenburg–Hirschsprung syndrome and WS type 4 [8]. WS4 is characterized by sensorineural hearing loss, depigmentation of hair, skin, and eyes, and Hirschsprung’s disease. The *SOX10* gene was first identified as the mutant gene responsible for megacolon and depigmentation in Dom mutant mice (SOX10*^Dom^*) [1,23]. Specifically, a frameshift mutation in *SOX10* causing haploinsufficiency was found to be the cause, with a homozygous mutation in mice proving lethal [24]. Based on this discovery, *SOX10* mutations were screened for in human patients with Waardenburg–Hirschsprung disease, in whom a causative mutation had not yet been identified. Several cases were found to have a *SOX10* mutation, confirming its involvement in the Waardenburg–Hirschsprung disease [8]. 

Waardenburg syndrome has been classified into four main presentations. Type I (WS1) presents with pigmentary abnormalities of the hair, heterochromia irides, sensorineural hearing loss, and the characteristic dystopia canthorum. Type 2 (WS2) has similar features with the absence of dystopia canthorum. Type 3 (WS3) is distinguished by abnormalities of the upper limb. While Waardenburg syndrome was initially classified by phenotypic presentation, detected mutations in patients with WS have been integrated into further subclassifications. For instance, WS4 has been split into WS4A, WS4B, and WS4C, with mutations in *EDNRB*, *EDN3*, and *SOX10*, respectively [25]. Another subtype, WS2E, is also caused by a *SOX10* mutation [26].

### 3.2. PCWH

PWCH (Peripheral demyelinating neuropathy, central demyelinating leukodystrophy, Waardenburg syndrome, and Hirschsprung disease) represents a neurological variant of the previously discussed WS4, where a *SOX10* mutation is also implicated. Patients with PWCH exhibit a similar presentation, including heterochromia irides, sensorineural hearing loss, and Hirschsprung’s disease, as observed in Waardenburg–Shah syndrome. Additionally, they experience neurological symptoms such as peripheral neuropathy, ataxia, and intellectual disability [27,28]. The syndrome was first described shortly after the discovery of *SOX10* mutations in WS4. Due to the shared features, mutations in the *SOX10* gene were investigated in patients with what is now termed PCWH. A de novo deletion mutation was identified in the coding region of *SOX10*, leading to an extension of the peptide and a toxic gain of function [29]. The discovery of a *SOX10* mutation as a perpetrator (and the exclusion of other known mutations such as *PMP22*) in PCWH, a demyelinating disease, further supported the role of *SOX10* in Schwann cell differentiation. 

### 3.3. Kallman Syndrome

Due to the presence of hypogonadism and anosmia in subtypes of Waardenburg syndrome (e.g., WS2E), *SOX10* was investigated as a potential candidate gene for Kallman syndrome (KS), which falls under the umbrella of congenital hypogonadotropic hypogonadism (HH). KS is characterized by anosmia, distinguishing it from idiopathic hypogonadotropic hypogonadism, which lacks anosmia. Both are considered manifestations of the same syndrome, and instances of each may coexist within the same family [30]. Although nine genes have been implicated in HH, demonstrating extensive genetic heterogeneity, they only account for 30% of KS cases. Therefore, *SOX10* appeared to be a likely candidate mutation to explain the presence of anosmia within the disease spectrum.

In a mouse model study, *SOX10* deficient mice exhibited an almost complete absence of olfactory ensheathing cells (OECs), misrouting of nerve fibers, impaired migration of GnRH cells, and disorganization of the olfactory nerve layer in the olfactory bulbs [31]. In the same study, a cohort of KS patients without known mutations were screened for *SOX10* mutations. Six patients had novel *SOX10* mutations, and five out of the six also had deafness. 

The diversity and overlap of clinical features in patients with Waardenburg–Shah syndrome, PCWH, and Kallman syndrome underscore the role of *SOX10* as a common factor for pathogenesis. However, the phenotypic variability among patients with the same mutations or in the same families emphasizes the need for further study of intermediate and downstream factors [3]. 

### 3.4. Hearing Loss

*SOX10* mutations in the inner ear explain abnormalities in hearing, such as hypoplasia of semicircular canals, enlarged vestibular canals, vestibulocochlear nerve agenesis, and cochlear deformities [32,33]. Although the presence of sensorineural hearing loss among patients with Waardenburg–Shah syndrome varied among genotypes, Song et al. found that the prevalence of hearing loss in patients with a *SOX10* mutation was 100% [33]. In mouse models, the expression of SOX10 in vestibulocochlear development has been studied, revealing an increase in SOX10 expression in the maturing cochleovestibular ganglion. In *SOX10*-deficient mice, there was a lack of glial cell development in this area [34]. Hearing loss is such a penetrant phenotype in patients with *SOX10* mutations that it can manifest without any other features of WS or KS, resembling isolated hearing loss [35].

As of yet, there is no clear role for *SOX10* in genetic screening or counseling for the discussed conditions. More effort is necessary to consolidate the range of phenotypes into one disease spectrum rather than individual syndromes. 

## 4. SOX10 Expression in Neural and Neuroectodermal Tumors

*SOX10*’*s* role is of interest in the development of certain malignancies and as a potential differentiating marker with diagnostic use. 

### 4.1. SOX10 Expression in Melanoma

The expression of SOX10 in melanoma has been conducted due to its significance in both diagnostic and therapeutic applications (Table 1). Bakos et al. investigated the expression of SOX10 through immunohistochemistry (IHC) in primary and metastatic melanoma cells and its association with neistin coexpression [36]. Nestin is an intermediate filament present in neural progenitor cells, melanomas, and melanocytic nevi. This study disclosed a significant co-expression of SOX10, SOX9, and neistin in early primary melanoma. However, no statistically significant co-expression was observed in the metastatic melanoma [36]. These results align with their in vitro findings, suggesting that SOX10 plays a crucial role in neistin activation during early melanoma development but is not associated with its expression in the more advanced stages of the disease [36]. These findings suggest that SOX10 may serve as a potential marker for determining melanoma stage.

In a separate study by Zhongyuan et al., the role of *SOX10* in melanoma development was similarly investigated. According to that study, *SOX10* plays an important role in regulating various factors involved in melanocyte proliferation and survival, including melanocyte inhibitory activity (*MIA*), *MITF*, *p21*/*WAF1*, and *E2F1* [37]. A reduction in SOX10 expression resulted in reduced melanoma formation, and the knockout of the *SOX10* gene led to the elimination of new tumor formation [37]. These findings provide additional evidence supporting the role of *SOX10* in melanocyte proliferation. That study also aimed to establish the downstream pathway through which *SOX10* affects melanocyte proliferation by observing its effects on the expression of the minichromosomal maintenance complex component (MCM5). The results demonstrated that the overexpression of *MCM5* in *SOX10*-negative cells partially rescued the proliferation defect observed when *SOX10* was absent [37]. Overall, these findings indicate that *SOX10* is involved in multiple melanocyte proliferation pathways, with the *SOX10*-*MCM5* axis playing a critical, though not exclusive, role in the proliferation [37].

Further evidence on the role of *SOX10* on melanoma cell proliferation was reported in a study by Cronin et al., which revealed that the loss of SOX10 in melanoma cells resulted in cell arrest in the G1 phase [38]. Molecular studies of melanoma cells with absent *SOX10* showed reduced expression of MITF, elevated expression of p21/WAF1 and p27KIP2, hypophosphorylated RB, and reduced levels of E2F1 [38]. These results suggest that the removal of *SOX10* leads to cell arrest in the G1 phase [38]. Another study by Rosenbaum et al. examined the role of *SOX10* in the regulation of the melanoma cell cycle, finding that knocking out *SOX10* in immune-competent models led to a reduced expression of immune checkpoint proteins HVEM and CEACAM1 [39]. The loss of these immune checkpoint proteins promotes the proliferation of malignant melanoma cells by preventing cellular senesce and apoptosis [39]. 

Studies on SOX10 have extended beyond its role in proliferation with investigations into its involvement in the migration of melanoma cells. Seong et al. explored this aspect by studying the migration of B16F10 melanoma cell lines following the introduction of siRNA specific for *SOX10*. This was compared to a control group of the same cell line. That study demonstrated a significant reduction in migration in the experimental cell line with downregulated *SOX10*, as confirmed through a TUNEL assay. Additionally, microarray screening revealed a three-fold decrease in *SOX10* and one of its downstream targets, *MITF* [40]. These findings highlight the significant role of *SOX10* expression and its effect on MITF in B16F10 melanoma cells, suggesting a crucial role in cell migration and, consequently, metastasis [40]. Attempts to replicate these results using different melanoma cell lines (Cloudman S9 and Melan-A melanoma cell lines) yielded no statistically significant effects on cell migration, emphasizing the variability of *SOX10* effects depending on the specific cell line being studied [40].

In light of the diverse yet persistent role of *SOX10* in melanoma cells, its potential as a diagnostic histopathological marker has been explored. Clevenger et al. conducted a comparative study using a pan-melanoma cocktail, a SOX10 stain, and an MITF stain to identify melanoma cells of epithelioid origin, those with a predominantly spindle appearance. That study revealed a 100% SOX10 positive staining pattern in both epithelioid and spindle-shaped cells, demonstrating nuclear staining with a strong and diffused pattern. In contrast, the pan melanoma cocktail and MITF stain showed positive staining in 86% and 93% of cases, respectively, for epithelioid cells, and 86% for spindle-shaped melanoma cells [41]. The high rate of detection using SOX10 staining suggests its utility in detecting metastasis in locations where a small number of cells would be expected, such as the cerebrospinal fluid (CSF). However, caution is advised, and a more sensitive stain for melanoma should be considered due to the non-exclusive expression of SOX10 [41]. 

*MITF*, downstream of the *SOX10* gene, plays a crucial role in the transcription control of melanocytes and retinal pigment cells and is strongly associated with malignancies [42]. Studies have shown that the absence or reduced activity of *SOX10* consistently leads to cell death in melanocytic descent, particularly at the G1 stage of the cell cycle. The impact on lineage is associated with the type of knockout, whether it involves a complete knockout or an interruption in the product’s structure, or a reduction in the half-life [39,43].

Notably, the knockout of *SOX10*, when simultaneously treating advanced melanoma, can confer resistive mechanisms against chemotherapeutic medications. Using Vemurafenib to treat advanced melanoma with an observed *BRAFV600E* mutation, cells acquiring a somatic *SOX10* mutation that hinders proper gene product formation allow the tumor to grow unchallenged by therapeutic treatments that would otherwise be effective [42,44]. This underscores the intricacies and complex integration of *SOX10*, which primarily directs proliferation and steers cells toward differentiated paths. Acting as an oversight system for downstream transcription factors, such as *MITF* [45], the gene gains unregulated function to promote transcriptive and translative efforts within the cell, allowing malignancies to establish their proliferative roots [39,44]. However, the knockout of *SOX10* in existing cancers can lead to acquired resistance against chemotherapeutic efforts. In other iterations of malignancies, knocking out the gene has been found to suspend cell proliferation, restrain cell growth, and reduce overall tumor size [39,42,43]. As melanomas approach their proliferative limits or the threshold for potential invasion, *SOX10* has been observed to become downregulated within the tumor cells. This change induces a phenotypic shift from melanocytic cell lineages to undifferentiated mesenchymal cell lines, characterized by their invasive nature and ability to resist targeted therapeutic regiments against malignancy [42].

Not all mutations of the *SOX10* locus are somatic. In studies focusing on childhood melanoma, almost all congenital melanomas were found to be *SOX10* positive. The significance of this positivity, whether it represents an unhindered function or a gain-of-function mutation, is yet to be determined. Regardless, its presence signifies its key integration in the early stages of skin neoplasms [46]. Studies supporting *SOX10* as a more sensitive marker for melanoma, compared to *MITF*, the previous standard marker for neoplastic testing within this sector, further highlight its diagnostic potential [3,45].

In research by Shakova et al. concerning the significance of SOX10 in melanoma and congenital giant melanocytic nevus, a pre-cancerous lesion heavily associated with melanoma formation, it was confirmed in mouse subjects and later human cell lines that the knockout of this transcription factor showed effective results in blocking tumorigenesis. Furthermore, the knockout or inactivation of the *SOX10* gene established its role as a prerequisite for the formation and maintenance of pre-melanoma lesions [46]. In observed human cell lines, the absence of *SOX10* activity resulted in an estimated nine-fold increase in apoptotic cells due to the disrupted regulation of apoptotic control factors. Examples of this dysregulation were noted from the increases in these control factors, such as caspases and proteins related to the tumor necrosis factor (TNF) pathway [46].

A study by Capparelli et al. demonstrated that *SOX10* plays a crucial role in mediating phenotypic switching in cutaneous melanoma. The loss of *SOX10* led to the development of an invasive, slow-cycling state in melanoma cells, promoting tolerance to *BRAF* and/or *MEK* inhibitors, which are commonly used in melanoma treatment. That study also identified a vulnerability in *SOX10*-deficient melanoma cells, specifically an up-regulation of cellular inhibitors of apoptosis-2 (*cIAP2*). The use of *cIAP1/2* inhibitors selectively induced cell death in *SOX10*-deficient cells, providing a potential therapeutic strategy to target and eliminate these cells. Additionally, combining *cIAP1/2* inhibitors with *BRAF*/*MEK* inhibitors delayed the onset of acquired resistance in melanomas in vivo [47]. 

### 4.2. SOX10 Expression in Malignant Peripheral Nerve Sheath Tumor and Schwannomas

While much of the existing data on the role of *SOX10* in neoplasms primarily focuses on melanoma, this gene is also implicated in other neural and neuroectodermal tumors. Malignant peripheral nerve sheath tumor (MPNST) is one such malignancy where the role of *SOX10* has been investigated. A study by Kang et al. aimed to assess SOX10 as a marker for distinguishing MPNST from synovial sarcoma, given the histopathological similarities that can make differentiation challenging [48]. SOX10 staining revealed a 67% positivity rate in MPNST cells compared to only 7% in synovial sarcomas. The overall results demonstrated a 67% sensitivity rate and a high specificity rate of 93% for SOX10 staining in MPNST, with a positive predictive value of 82% and a negative predictive value of 89% [48]. These findings suggest that SOX10 staining is moderately sensitive but highly specific, serving as a valuable marker for differentiating MPNST from synovial sarcomas in cases where there is a diagnostic discrepancy [48].

Another study by Pekmezci et al. investigated the use of SOX10 as a differentiating marker between MPNST and schwannomas, revealing a positive diffuse SOX10 expression pattern seen only in cellular schwannomas [49]. The results imply that SOX10 expression is significantly more prevalent in cellular schwannomas, and its loss of expression is indicative of MPNST when compared to cellular schwannomas [49]. Doddrell et al. explored SOX10 expression in merlin-null schwannomas, finding reduced expression of SOX10 and two proteins crucial for the myelinating function of Schwann cells: KROX20 and OCT6 [50]. Reintroducing the *SOX10* gene in schwannoma cells showed a small increase in KROX20 expression, which significantly increased with the introduction of cAMP [50]. Overall, the results suggest that the loss of *SOX10* in Schwann cells leads to cellular abnormalities resembling schwannomas [50]. Collectively, these studies indicate that SOX10 expression is a relatively effective marker for differentiating between specific malignancies that may pose diagnostic challenges. Moreover, a recurring pattern in the reported results suggests that SOX10 expression tends to decrease as cells undergo a transition from normal to malignant states in tumors.

**Table 1:** Studies demonstrating SOX10 expression in neural and neuroectodermal tumors.

Tumor	References	Findings
Melanoma	[51]	Role of SOX10 in Melanoma: *SOX10* serves as a crucial regulator of melanoma invasion and survival by influencing the expression of key factors such as MIA (melanocyte inhibitory activity), MITF, p21/WAF1, and E2F1; The absence of SOX10 expression has been linked to a reduction in melanoma formation, and silencing *SOX10* leads to the elimination of tumor formation in vivo; *SOX10* plays a pivotal role in regulating melanocyte proliferation through its interaction with the minichromosomal maintenance complex component 5 (MCM5); Inducing overexpression of MCM5 in *SOX10* knockout cells partially rescues the cell’s impaired proliferation capacity.
	[45]	Loss of *SOX10* in melanoma cells results in cell cycle arrest in the G1 phase, accompanied by molecular changes such as reduced MITF expression, elevated p21/WAF1 and p27KIP2 expression, hypo-phosphorylated RB, and reduced levels of E2F1; *SOX10* is essential for melanogenesis.
	[39]	*SOX10* knockout in immune-competent models leads to reduced expression of immune checkpoint proteins HVEM and CEACAM1, facilitating tumor growth.
	[40]	SOX10 in Melanoma Cell Migration and Metastasis: siRNA specific for *SOX10* demonstrates that downregulation of *SOX10* in B16F10 melanoma cells significantly reduces cell migration compared to control cells in a Transwell migration assay; TUNEL assay results indicate that the lower migration in the experimental cell line is not due to apoptosis or senescence; Microarray screening reveals a three-fold decrease in *SOX10* and in *MITF*, a known target of *SOX10*; The gene expression cascade initiated by *SOX10* and mediated by *MITF* plays a significant role in melanoma cell migration and metastasis; These effects are not reproducible in Cloudman S9 and Melan-A cells, suggesting that the SOX10/MITF effects on migration and metastasis vary depending on the melanoma cell line.
	[41]	SOX10 as a Diagnostic Marker for Melanoma: SOX10 staining is highly effective in identifying melanoma cells, with a 100% positivity rate in both epithelioid melanoma and melanoma with a predominantly spindle cell appearance; Given its high detection rate and strong staining intensity, SOX10 is a valuable marker for detecting melanoma cell metastasis in locations like the cerebrospinal fluid (CSF), where a large number of cells are not expected. However, a more sensitive melanoma stain should be used for confirmation, considering SOX10 is not exclusively specific for melanoma
	[36]	SOX10 and Nestin in Melanoma Development: Nestin, an intermediate filament found in neural progenitor cells, melanomas, and melanocytic nevi, shows statistically significant co-expression with SOX10 in primary melanomas; SOX10 plays a key role in Nestin activation in primary melanoma cells, suggesting that SOX10 is a major mediator of early melanoma development.
Malignant peripheral nerve sheath tumor	[48,49]	SOX10 in Differentiating MPNSTs and Synovial Sarcomas: SOX10 staining demonstrates a 67% positivity rate for MPNST, compared to only 7% in synovial sarcomas; SOX10 staining exhibits a sensitivity of 67% and specificity of 93%, making it a moderately sensitive but highly specific marker for distinguishing MPNST from synovial sarcoma.
Merlin-null schwannoma	[50]	SOX10 in Schwannomas and Normal Schwann Cell Function: Loss of SOX10 expression strongly supports the diagnosis of MPNST; Schwannoma cells show reduced SOX10 expression, as well as diminished expression of KROX20 and OCT6, crucial proteins in the myelination process; Reintroduction of the *SOX10* gene in schwannoma cells increases KROX20 expression, particularly in the absence of cAMP, with a significant boost upon cAMP introduction; Removal of *SOX10* from normal Schwann cells in a mouse model results in minimal expression of KROX20 and OCT6, irrespective of cAMP levels; SOX10 expression is necessary for normal Schwann cell function, and its loss leads to abnormalities resembling those seen in schwannoma cells.

## 5. SOX10 Expression in Mesenchymal Tumors

In a study conducted by Miettinen et al., the expression of SOX10 was analyzed in 1645 non-neurogenic mesenchymal tumors. Among non-nerve sheath tumors, positive SOX10 tumor cells were identified only in alveolar rhabdomyosarcoma (2/27) and ossifying fibromyxoid tumors (2/47). Thirty-three other types of mesenchymal tissues analyzed (1571 samples), including fibroblastic-myofibroblastic tumors, benign fibrous histiocytoma and subtypes, solitary fibrous tumor/hemangiopericytoma of the peripheral soft tissues and intracranial space, and undifferentiated pleomorphic sarcomas, were negative for SOX10. Synovial sarcomas, desmoid fibromatosis, and glomus tumors showed fewer than 5% of SOX10-positive nuclei, possibly representing entangled neural components [52].

Research by Karamchandani et al. aimed to validate the use of SOX10 and S100 protein as reliable markers in soft tissue neoplasms of both neural crest and non-neural crest origin. SOX10 and S100 mRNA levels were evaluated in 122 cases of peripheral nerve sheath tumors and synovial sarcomas, and IHC was used for SOX10 and S100 protein expression in 1012 tissue specimens [53]. Synovial sarcomas expressed significantly higher levels of S100 than SOX10, and no significant SOX10 mRNA expression was identified in synovial sarcoma [53]. The majority of schwannomas and neurofibromas showed increased expression of both SOX10 and S100 mRNA [53]. MPNSTs revealed highly correlated, variable levels of SOX10 and S100 mRNA expression. Of the non-neural, nonmelanocytic sarcomas, only one rhabdomyosarcoma sample was positive for SOX10. In summary, SOX10 was positive in only 5 of 668 cases with a 99% specificity for non-schwannian, nonmelanocytic tumors [53].

Kang et al. evaluated the diagnostic utility of SOX10 IHC in differentiating between synovial sarcoma and MPNST due to similar histomorphology and immunophenotype [48]. Forty-eight cases of MPNST and 97 cases of synovial sarcoma, including four intraneural synovial sarcomas, were stained for SOX10. Sixty-seven percent of MPNST (32/48) and only 7% (7/97) of synovial sarcomas were positive for SOX10. Nevertheless, there is uncertainty as to whether SOX10-positive cells in intraneural synovial sarcoma represent entangled Schwann cells, synovial sarcoma cells, or both [48]. 

In an attempt to demonstrate the clinical and morphological heterogeneity between gastrointestinal mesenchymal tumors with neurotrophic tyrosine receptor kinase (*NTRK*) gene rearrangements and gastrointestinal stromal tumors, Atiq et al. reported consistently absent SOX10 expression in eight mesenchymal tumors in the gastrointestinal tract with *NTRK1* or *NTRK3* rearrangements [54].

Research by Chiang et al. focused on classifying a newly discovered category of high-grade uterine sarcomas. Four *NTRK* fusion-positive uterine sarcomas were identified and distinguished from both undifferentiated uterine sarcomas and more commonly aggressive leiomyosarcomas. All four mesenchymal tumors lacked SOX10 expression [55].

## 6. SOX10 Expression in Epithelial Neoplasms

When examining the impact of SOX10 on epithelial neoplasms, its influence is extensive and continues to unfold with further investigations. This transcription factor plays a crucial role in regulating the proliferation and specialization processes of melanocyte and Schwannian lineages, exhibiting high expression levels in melanoma malignancies and those affecting the central nervous system [3]. While many of the studied mutations indicate somatic changes, there are instances of inherited cases [46]. In the observed cases, SOX10 expression is more prevalent in malignancies during proliferative stages compared to those found in invasive or metastatic stages [44] (Table 2).

Beyond tumors involving melanocytic lineages, research has provided substantial evidence of SOX10 expression in the salivary gland, breast, and ovarian neoplasms affecting epithelial cells. Although this evidence has accumulated in recent years, sensitivity for diagnostic differentiation, particularly in salivary gland tumors, remains less reliable [56]. Conversely, concerning ovarian cancers, distinguishing between SOX10 expression within the nucleus and cytoplasm has shown promise in estimating grade and prognosis.

In the context of salivary gland neoplasms, SOX10 expression has been identified in tumors arising from acinar and intercalated ductal cells [57,58,59]. Notably, tumors lacking SOX10 have been associated with the appearance of excretory ducts or striated ducts [59]. SOX10-expressing neoplasms in the salivary glands include acinic cell carcinoma, epithelial-myoepithelial carcinoma, adenoid cystic carcinoma, and polymorphous adenocarcinoma [59,60]. Adenoid cystic carcinoma and polymorphous adenocarcinoma have consistently demonstrated SOX10 expression in virtually all cases studied [57,58]. Distinctively, acinic cell carcinoma can be differentiated from metastatic renal cell carcinoma in the parotid gland, as the latter does not express SOX10 on staining [58].

However, certain salivary gland neoplasms either show no SOX10 representation or exhibit focal expression in staining. These include salivary duct carcinoma, mucoepidermoid carcinoma (MEC), squamous cell carcinoma (SCC), and oncocytic carcinoma, which originate from excretory and serous ductal cells within the salivary glands. While MEC tumors were initially considered SOX10-negative, further investigation revealed a subgroup of SOX10-positive MEC with distinct morphology and colloid-like secretion [57,59]. Additionally, SOX10 has been found to be positive in other tumors, such as basal cell carcinomas (BCCs) and low-grade salivary duct carcinomas [57]. In the case of SCC secondary to HPV infection, SOX10 is not a reliable diagnostic marker due to similar staining distributions with HPV-related multiphenotypic sinonasal carcinoma [61]. While SOX10 staining can aid in categorizing tumors based on cell origins, negative staining does not necessarily imply the absence of SOX10 mutation, as inactivating or truncating mutations can result in reduced or absent SOX10 expression [59,61,62]. Despite this, SOX10 is considered a valuable protein expression marker for the diagnostic identification of salivary gland neoplasms, contributing to increased diagnostic accuracy [58].

SOX10 protein expression has also been observed in breast carcinomas, particularly in approximately 66–74% of triple-negative breast carcinomas [63]. Triple-negative breast carcinoma has shown SOX10 expression in a substantial number of cases, ranging from 38 to 67% in the literature [62]. SOX10 has been associated with CD117 and vimentin expression in triple-negative breast carcinomas, though its prognostic value remains inconclusive and is mainly considered a marker for aiding in differential diagnoses [62]. While evidence suggests a potential prognostic value due to associations with malignant characteristics of triple-negative breast carcinomas, further research is needed to establish its definitive prognostic significance [64]. In cases where homozygous deletions and point mutations eliminate SOX10 staining presence, GATA3, a common marker in breast carcinoma, has been used in conjunction with SOX10 to address this limitation. Approximately 60% of triple-negative breast carcinomas have been identified using this dual-staining method, making SOX10 a useful marker in identifying epithelial neoplasms of the breast [62,65]. 

In the context of ovarian epithelial tumors, SOX10 has shown value in differentiating cell origin and estimating prognosis. Contrary to previous claims that suggested no application for SOX10 in the study and diagnosis of ovarian epithelial tumors, Kwon et al. demonstrated its utility. Ovarian epithelial neoplasms, including serous, mucinous, and endometrioid subtypes, can be differentiated based on the localization of staining. Serous neoplasms show nuclear localization, while mucinous and endometrioid neoplasms exhibit cytoplasmic localization [66]. Staining in both regions is possible, but the diffuse characteristic of SOX10 staining helps distinguish between subtypes. The intensity of the stain within the nucleus correlates with the prognosis of the patient, emphasizing its potential as a prognostic marker [66]. While SOX10’s involvement in ovarian carcinomas was assessed, other common expression markers studied for ovarian cancer include SOX8 and, notably, SOX9, which has been implicated in various signaling pathways in ovarian cancer development [67,68,69,70,71]. 

In nasopharyngeal carcinomas, SOX10 is markedly overexpressed, and this overexpression is associated with a poorer prognosis, particularly in T classification and lymph node metastasis. The correlation with poor prognosis is linked to SOX10’s involvement in tumor development and metastatic-seeding ability in breast cancer cells. The overexpression of SOX10 in nasopharyngeal tumors highlights its potential importance as a diagnostic and prognostic marker for patients with nasopharyngeal carcinoma [72]. 

SOX10 has also been associated with metaplastic bladder cancers, where it exhibits elevated expression in bladder cancer tissues compared to healthy tissue. Knockdown experiments targeting *SOX10* confirmed its prognostic value by significantly impacting the growth and metastatic ability of bladder cancer. The suspected mechanism involves SOX10 influencing the expression of other components such as B-catenin and Met. Targeting SOX10 as a marker for diagnosis, prognosis, and treatment may prove useful in the context of bladder cancer [16].

In summary, while further research is needed to fully understand the extent of SOX10 expression in various epithelial neoplasms, it remains a promising marker for the diagnosis and prognosis development of several carcinomas. Additionally, it shows potential as a treatment target in certain cancers.

**Table 2:** SOX10 expression in epithelial neoplasms.

Epithelial Neoplasm	SOX10 Expression	Implications
Ovarian serous, mucinous, and endometrioid carcinoma	Overexpressed	SOX10 exhibits stem cell-supporting properties in both normal and cancerous cells [66]; SOX10 presence is associated with chemoresistance, possibly contributing to poorer prognoses in certain cancers [66]; SOX10 helps differentiate cell origin and estimate prognosis in ovarian epithelial tumors; Subtypes such as serous, mucinous, and endometrioid can be differentiated based on the localization and intensity of SOX10 staining [66].
Triple-negative breast cancer	Overexpressed	Clinical significance of SOX10 in breast carcinomas is not fully understood, but it is considered a useful marker [62]; Approximately 66–74% of triple-negative breast carcinomas express SOX10 [63]; Prognostic value is unclear, but there is evidence suggesting a possible contribution to malignant characteristics [62]; High sensitivity in identifying triple-negative breast carcinomas [62,63,64].
Nasopharyngeal carcinomas	Overexpressed	Marked overexpression of SOX10 is observed in nasopharyngeal carcinomas [72]; Higher expression is associated with a poorer prognosis, and SOX10 is believed to be involved in tumor growth and metastasis [72]; Potential importance as a diagnostic and prognostic marker [72].
Bladder carcinomas	Overexpressed	SOX10 is significantly elevated in bladder carcinomas compared to surrounding healthy tissues [16]; Knockdown of *SOX10* impacts cancer growth and spread, making it a potential treatment target [16]; SOX10 inhibition may affect cancer progression by influencing other components in development pathways such as B-catenin and Met [16]; Potential usefulness as a diagnostic marker for bladder cancers [16].
Salivary gland neoplasms	Overexpressed	SOX10 helps distinguish between various types of salivary gland neoplasms [57]; SOX10 rules out mimic lesions, differentiates between high- and low-grade adenocarcinomas, and is a reliable marker against certain similar-appearing tumors [57]; Tumors lacking SOX10 are associated with specific histological features, such as the appearance of excretory or striated ducts; SOX10 expression varies among different subtypes of salivary gland neoplasms [57,58,59].
Gastrointestinal Mesenchymal Tumors	Lost	SOX10 expression is absent or minimal in gastrointestinal mesenchymal tumors with *NTRK* gene rearrangements, distinguishing them from gastrointestinal stromal tumors [54].
Uterine Sarcomas	Lost	SOX10 lacks expression in a category of uterine sarcomas with NTRK fusions, distinguishing them from undifferentiated uterine sarcomas and aggressive leiomyosarcomas [55].

## 7. Expression of Other Members of the “SRY-Related HMG Box” in Cancers

### 7.1. The HMG Box Family

The HMG box is a versatile protein domain consisting of about 75 amino acids that plays a crucial role in DNA binding and various transcription and translation processes. The name “High Mobility” originates from the initial discovery of these proteins in the acid extracts of mammalian chromatin, where they exhibited significant electrophoretic mobility [73]. 

HMG box domains can be broadly categorized into two types based on their DNA binding specificity: non-sequence specific; and sequence specific [74]. Both types of HMG box domains exhibit a high affinity for non-B-type DNA structures, which include bent, kinked, and unwound DNA. Additionally, these domains are involved in diverse protein-protein interactions, such as DNA bending, looping, and unwinding [74,75].

#### 7.1.1. Non-Sequence Specific HMG Box Domains

- Proteins in this category, such as HMGB1-4, typically possess two HMG boxes or four to six HMG boxes in the presence of transcription factor UBF [75];
- Mammals have four HMGB proteins (HMGB1-4), and they function as DNA chaperones, contributing to processes like transcription and DNA repair. However, each of these proteins has distinct characteristics [75].

#### 7.1.2. Sequence Specific HMG Box Domains

- Proteins classified as sequence-specific usually have a single HMG box and lack acidic C-tails, which are common in non-sequence-specific HMG box proteins [74];
- Examples of proteins in this category include TCF, SRY, and SOX [75];
- Despite recognizing specific DNA sequences, these proteins form few base-specific hydrogen bonds, resulting in less sequence specificity [75].

### 7.2. SRY-Related HMG Box

The *SOX* genes, a subset of HMG box-type proteins, are encoded by 20 different genes in both humans and mice. These genes, located within the *SRY* gene on the Y chromosome, play pivotal roles in various cellular processes, including stemness maintenance, cell lineage determination, differentiation, proliferation, and even cell death. Unlike typical DNA modification mechanisms, *SOX* genes achieve their functions by binding specifically to the minor groove of pre-existing DNA, thereby influencing its shape and facilitating higher affinity binding of DNA to various transcription factors [76]. Key features of the *SOX* genes include the below.

#### 7.2.1. Genetic Organization

- *SOX* genes are organized into eight groups (A–H), with group B further divided into subgroups B1 and B2 [76];
- Within the same group, SOX proteins share a high degree of structural and identity similarity, ranging from 70% to 95%, both in the HMG box domain and in external characteristics;
- Groups outside the same group have partial similarities in identities (>46%) in the HMG box domain and none in the external domains [76];

#### 7.2.2. Functions and Mechanisms

- *SOX* genes play crucial roles in DNA replication and mutations, contributing to diverse cellular processes [76];

#### 7.2.3. Individual SOX Genes

- The specific locus and schematic of the different *SOX* genes are detailed in Table 3

The following sections will provide insights into the implications of individual *SOX* gene groups in the genesis and progression of common cancers. The diversity within the *SOX* gene family allows for a wide range of functions and regulatory roles in cellular processes, making them essential players in normal development as well as potential contributors to cancer development [76].

**Table 3:** Specific locus and schematic of the different *SOX* genes. The blue oval represents the HMG box domain. The text within the blue oval indicates which SOX gene the schematic correlates to. The hexagon with “TA” indicates a transactivation domain. The hexagon with “TR” indicates a trans-repression domain. The gray diamond with “D” indicates a dimerization domain. Schematics were created with BioRender.com (2023).

Group	Gene	Locus	Schematic
A	*SRY*	YC3	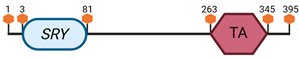
B1	*SOX1*	8 A1–A2	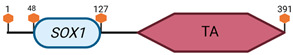
	*SOX2*	3 A2–B	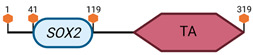
	*SOX3*	X A7.3–B	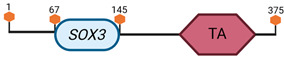
B2	*SOX14*	9 E3.3	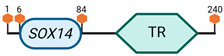
	*SOX21*	14 E4	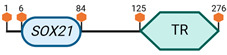
C	*SOX4*	13 A3–A5	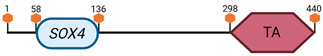
	*SOX11*	12 A3	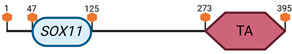
	*SOX12*	2 G3	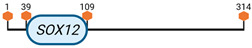
D	*SOX5*	6 G3	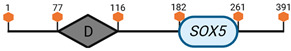
	*L-SOX5*	6 G3	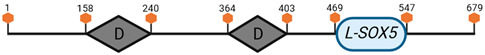
	*SOX6*	7 F1	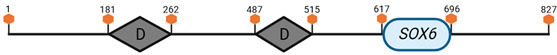
	*SOX13*	1 E4	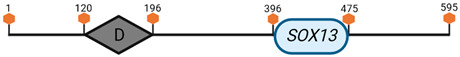
E	*SOX8*	17 A3	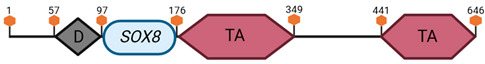
	*SOX9*	11 E2	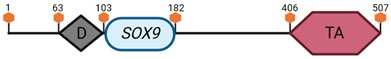
	*SOX10*	15 E1	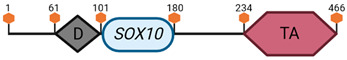
F	*SOX7*	14 C3	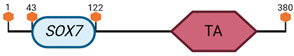
	*SOX17*	1 A1	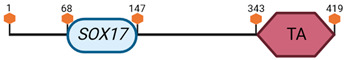
	*SOX18*	2 H4	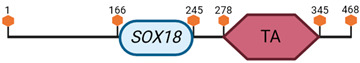
G	*SOX15*	11 B3	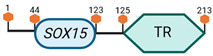
H	*SOX30*	11 B1.1	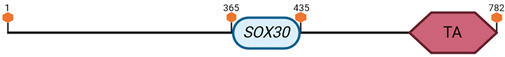

##### Group A

Group A of the *SOX* gene family consists of a single member, *SRY* (Sex-determining Region Y), and its primary function is to determine sex in mammals [77,78]. In the context of cancer, particularly in prostate cancer, the role of *SRY* is not well understood, and it is unclear whether *SRY* acts as a tumor suppressor or has other implications in cancer development [79]. Downregulation of *SRY* has been observed in prostate cancer, but it often occurs concurrently with the downregulation of other Y chromosome-specific genes [79]. Therefore, it would be premature to attribute the development and proliferation of prostate cancer solely to the downregulation of *SRY*. Further research is needed to elucidate the specific role of *SRY* and its potential contributions to prostate cancer and other cancers.

##### Group B (B1 + B2)

Group B of the *SOX* gene family consists of *SOX1*, *SOX2*, *SOX3*, *SOX14*, and *SOX21*. *SOX1*, *SOX2*, and *SOX3* belong to subgroup B1, while *SOX14* and *SOX21* fall into subgroup B2 [77,80,81,82].

***SOX1***

- Function: *SOX1* plays a crucial role in maintaining stem cell lineage, particularly in embryogenesis, differentiation, and mammalian brain development. It is essential for the survival and function of dopaminergic neurons [80];
- Oncogenic properties: *SOX1* has been implicated in the development of small cell lung, central nervous system, breast, and ovarian cancers. In small-cell lung cancer, *SOX1* collaborates with *NKX2*.*1* to maintain its identity and function. In central nervous system tumors like glioblastomas, *SOX1* extends the survivability of cancer cells [83]. In breast and ovarian cancer, *SOX1* acts as a tumor suppressor by inhibiting the Wnt/B-Catenin and *STAT3* signaling pathways [84,85];

***SOX2***

- Function: *SOX2* is a transcription factor that prolongs stemness in both embryonic and adult stem cells [86];
- Oncogenic properties: Dysregulation of *SOX2* expression is associated with increased proliferation and metastasis in the central nervous system and lung carcinomas [86];

***SOX3***

- Function: *SOX3* is upregulated in esophageal SCC, ovarian carcinoma, and osteosarcoma, promoting proliferation and migration [87]. It induces apoptosis in human breast cancer cell lines [87];

***SOX14***

- Function: *SOX14* is involved in the development of cervical cancer, inducing *P53* activation, which leads to apoptosis in cervical carcinoma cell lines [88]. It also promotes proliferation and invasion through the Wnt/B-catenin pathway [88,89];

***SOX21***

- Function: *SOX21* has a tumor suppressor-like function in central nervous system cancers [90], inhibiting the carcinogenic properties of *SOX2* [91]. Forced expression of *SOX21* induces cellular apoptosis in glioma cells and enables differentiation, preventing glioma formation [90].

These *SOX* genes in Group B exhibit diverse functions and play critical roles in various cancers, either promoting or inhibiting oncogenic processes. Their involvement underscores the complexity of *SOX* gene functions in different cellular contexts and cancer types;

##### Group C

The *SOX* genes that have been classified into group C include *SOX4*, *SOX11*, and *SOX12*;

***SOX4***

- Function: *SOX4* is implicated in embryogenesis and tissue development [92,93,94,95,96,97];
- Cancer associations: Elevated *SOX4* expression is observed in leukemia, colorectal, lung, breast, and hepatocellular cancers [92,93,94,95,96,97]. In hepatocellular carcinoma, increased *SOX4* expression inhibits *P53*-directed apoptosis by restricting *BAX* expression [96];

***SOX11***

- Function: *SOX11* serves as both a causative and protective agent in various tumors;
- Cancer associations: Upregulation of *SOX11* is seen in medulloblastoma, mantle cell lymphoma, endometrial and breast cancer, Burkitt’s lymphoma, colorectal cancer, lung adenocarcinoma, lung SCC, and ovarian cancer [98,99,100,101,102,103,104]. *SOX11* expression is a unique feature in certain cancers and helps distinguish them from other malignancies [105,106];
- Prognostic factors: High *SOX11* expression in gastric and ovarian cancers is linked to higher survival rates, while in breast cancers, the opposite is observed [102,107];

***SOX12***

- Function: Hepatocellular carcinomas positive for *SOX12* exhibit increased proliferation, malignant potential, and higher resistance to cisplatin, a common chemotherapy agent [108];
- Cancer associations: *SOX12* is involved in gastric, lung, hepatocellular, colorectal, renal carcinomas, and thyroid cancers (elevated levels) [51,108,109,110]. Increased *SOX12* expression in thyroid cancer cells is associated with promoting carcinogenic properties [51].

The *SOX* genes in Group C play diverse roles in embryonic development and tissue maintenance and are implicated in various cancers. They contribute to the complexity of cancer biology by either promoting or inhibiting tumorigenesis depending on the specific context and cancer type;

##### Group D

The *SOX* genes that have been classified into group D include *SOX5*, *SOX6*, and *SOX13*;

***SOX5***

- Function: *SOX5* plays a role in the development and differentiation of embryonic germ cell lines [111];
- Cancer associations: Similar to other *SRY*-related HMG box genes, *SOX5* elevates the ability of cancer to grow, metastasize, and invade through angiogenesis. It is implicated in hepatocellular, breast, and gastric cancer [112,113,114];
- Unique properties: *SOX5* can mediate the epithelial-to-mesenchymal transition (EMT), a fundamental process in metastasis, by regulating the expression of E-cadherin and vimentin [112,115,116];

***SOX6***

- Function: *SOX6* exhibits both tumor suppressor and oncogenic properties depending on the cancer type;
- Cancer associations: *SOX6* is downregulated in osteosarcoma [117], esophageal SCC [118], hepatocellular carcinoma [119], and pancreatic β-cell cancers [120]. It shows oncogenic properties in gliomas [121] and endometrial cancers [122];
- Unique properties: *SOX6* induces autophagy in cervical cancer cell lines, leading to increased resistance to cisplatin chemotherapy and enhanced survivability [123];

***SOX13***

- Cancer associations: *SOX13* is highly expressed in oligodendrogliomas, gliomas, gastric carcinomas, and hepatocellular carcinomas [123,124,125,126]. *SOX13* overexpression in hepatocellular carcinoma activates *TWIST1*, a major transcription factor in embryonic development, promoting cancer metastasis [126]. *SOX13* supports stem-like properties in hepatocellular carcinoma, contributing to increased self-renewal, resistance to chemotherapy, and tumorgenicity [127].

These *SOX* genes, namely *SOX5*, *SOX6*, and *SOX13*, demonstrate diverse roles in embryonic development and cancer biology. Their involvement in processes like EMT, autophagy induction, and support for stem-like properties highlights their significance in the complex landscape of cancer progression and metastasis;

##### Group E

The *SOX* genes that have been classified into group E include *SOX8*, *SOX9*, and *SOX10*;

***SOX8***

- Function: *SOX8* has some minor effects on the specification and differentiation of glial cells;
- Cancer associations: *SOX8* expression is greatest during central nervous system development in immature cells. Elevated levels of *SOX8* indicate an undifferentiated state in the gliomas [124];

***SOX9***

- Function: *SOX9* is involved in multiple cancers in a variety of ways;
- Cancer associations: In some breast cancer subtypes, *SOX9* is involved in a positive feedback loop through Wnt/β-catenin activation [128]. Prostate cancer tends to be correlated with elevated levels of SOX9 [129]. *SOX9* contributes to cell proliferation and invasion in renal cell carcinoma. MiRNA-138-induced *SOX9* suppression prevents renal cell carcinoma progression [130]. Through the WNT/β-catenin pathway, *SOX9* is involved in cancer cell proliferation and invasion in papillary thyroid cancer [131]. *SOX9* increases *LGR5* expression, imparting the ability of glioblastoma cells to undergo tumorigenesis [77]. Elevated levels of *SOX9* expression in colorectal cancers are associated with lower 5-year survival rates [132]. *SOX9* levels are increased in non-small lung cancer [77] due to tumor-associated macrophages, which release TGF-β [133]. In skin cancers, *SOX9* levels are elevated too [77]. Increased *SOX9* levels cause melanoma cells to metastasize [134]. *SOX9*-involved keratinocyte proliferation also occurs in cutaneous BCC and cutaneous SCC [135].

*SOX8*, *SOX9*, and *SOX10* play diverse roles in different cancers, influencing processes such as differentiation, proliferation, invasion, and tumorigenesis. Understanding their specific functions in various cancer types is crucial for developing targeted therapeutic approaches;

##### Group F

The *SOX* genes that have been classified into group F include *SOX7*, *SOX17*, and *SOX18*;

***SOX7***

- Cancer associations: *SOX7* is implicated in several cancers. In breast cancer, *SOX7* functions as a tumor suppressor [136]. Hypermethylation-mediated silencing of the *SOX7* promoter is associated with greater carcinogenesis in breast cancer [136]. *SOX7* can be used as a marker for prognosis in prostate cancer. Its downregulation may be involved in the castration-resistant progression of prostate cancer [129]. *SOX7* also exhibits tumor-suppressive effects in gastric cancer through potential involvement in abnormalities with the *SOX7*-associated WNT/β-catenin pathway [137]. *SOX7’s* tumor suppressor effects have also been delineated in non-small-cell lung cancer, targeted by microRNA-9 [138];

***SOX17***

- Cancer associations: *SOX17* is associated with several cancers. Hypermethylation-dependent silencing of the *SOX17* promoter may induce inappropriate activation of the Wnt pathway, giving rise to breast cancer, thyroid cancer, gliomas, and gastrointestinal tumors [139,140,141,142]. Melanoma pathogenesis is also associated with decreased SOX17 expression; however, the mechanism is unclear [143];

***SOX18***

- Function: *SOX18* takes part in the development of blood and lymphatic vessels, as well as hair follicles [144]. Wound healing also involves SOX18 [145].
- Cancer associations: *SOX18* is associated with breast, lung, and skin cancers. In breast cancer, there is a positive correlation between *SOX18* and vascular endothelial growth factor D (*VEGF-D*), suggesting that *SOX18* positively influences angiogenesis [144]. In non-small-cell lung cancer, *SOX18* expression is noted in cells and vessels, and its expression may be used as a prognostic marker [145]. In skin cancers, elevated *SOX18* expression is involved in the formation of BCC and SCC [146].

Understanding the roles of *SOX7*, *SOX17*, and *SOX18* in various cancers provides insights into their potential as diagnostic markers and therapeutic targets in cancer treatment;

##### Group G

The sole member of this group is *SOX15*. Compared to the other members of the *SOX* family, it has been relatively understudied. Overexpression of *SOX15* is linked to lower proliferation of testicular embryonic cancer cell lines [147]. *SOX15* serves as a potential tumor suppressor gene and is negatively associated with the development of pancreatic ductal adenocarcinoma through the Wnt/B-catenin pathway [148]. Additionally, *SOX15* is repeatedly underexpressed among cancer cell lines, including colon, prostate, stomach, and uterine cancers, and overexpressed in some subsets of lung carcinomas [149].

*SOX15*, despite being relatively understudied compared to other *SOX* family members, demonstrates potential significance in regulating proliferation and acting as a tumor suppressor in specific cancer types, such as testicular embryonic cancer and pancreatic ductal adenocarcinoma. Its differential expression across various cancers suggests a context-dependent role, and further research may unveil its precise mechanisms and therapeutic implications;

##### Group H

*SOX30* is the sole member of Group H [77]. It acts as a tumor suppressor by activating *P53* transcription, leading to apoptosis. *SOX30* inhibits T-cell factor (TCF) either by binding to β-catenin or inhibiting β-catenin transcription [77,150,151]. Regarding lung adenocarcinoma specifically, the latter can be associated with hypermethylation of the *SOX30* gene. *SOX30’s* inhibition of TCF can contribute to the development of lung cancer. It also functions as a tumor suppressor by activating desmosomal genes, impeding cancer growth and spread [77,152].

## 8. Conclusions and Future Directions

In conclusion, *SOX10* emerges as a pivotal transcription factor with a multifaceted role extending from embryonic development to the pathogenesis of diverse pathological conditions. Its critical significance is exemplified by its association with congenital disorders such as Waardenburg–Shah Syndrome, PCWH syndrome, and Kallman syndrome, where mutations disrupt neural crest development. Within neural and neuroectodermal tumors, *SOX10* serves as a key player influencing proliferation and differentiation, making it a promising diagnostic and therapeutic marker.

The spotlight on *SOX10* intensifies in melanoma, where its impact on crucial factors like MITF and cell migration shapes tumor progression and treatment responses. In mesenchymal tumors, *SOX10* expression becomes a valuable tool for distinguishing between different tumor types, thereby facilitating accurate diagnoses and informed treatment decisions.

Epithelial neoplasms further underscore *SOX10’s* clinical relevance. Its expression or absence provides crucial insights into tumor cell origins, prognosis, and treatment responses. Particularly in ovarian cancer, *SOX10’s* involvement in chemoresistance highlights its significance in clinical settings.

The multifunctionality of *SOX10* positions it as a promising candidate for extensive research and clinical applications across various pathological conditions. As we delve deeper into its intricacies, there is potential for improved diagnostic accuracy and the development of more effective therapeutic strategies. *SOX10* stands at the intersection of basic research and clinical utility, holding promise for advancements that could reshape our approach to a spectrum of diseases.

## Data Availability

No new data were created or analyzed in this study. Data sharing is not applicable to this article.
